# Addressing barriers to global multidisciplinary stakeholder inclusivity: Lessons from global orofacial cleft research priority setting

**DOI:** 10.7189/jogh.14.04261

**Published:** 2024-12-13

**Authors:** Niki Kouvroukoglou, Sanita Sandhu, Barbara Delage, Debbie Sell, Nicola Stock, Gareth Davies, Marina Campodonico, Bruce Richard, Zipporah Njeri Gathuya, Mekonen Eshete, Felicity V Mehendale

**Affiliations:** 1 Medical School, University of Edinburgh, Edinburgh, UK; 2 The London Clinic, London, UK; 3 Smile Train, New York, USA; 4 Centre for Outcomes and Experience Research in Children’s Health, Illness and Disability (ORCHID), Great Ormond Street Hospital for Children NHS Foundation Trust and Speech@Home, London, UK; 5 Centre for Appearance Research, University of West England Bristol, Bristol, UK; 6 European Cleft Organisation, Rijswijk, The Netherlands; 7 Fundacion Grantz, Santiago de Chile, Chile; 8 Cleft surgeon, Birmingham, UK; 9 The Nairobi Hospital, Nairobi, Kenya; 10 Cleft Lip and Palate Program Yekatit 12 Hospital Medical College, Addis Ababa, Ethiopia; 11 Global Cleft Research Programme, Usher Institute, University of Edinburgh, Edinburgh, UK

## Abstract

**Background:**

Inclusivity in research priority setting is fundamental to capturing the opinion of all stakeholders in a research area. Globally, experienced healthcare workers often have deep insights that could impactfully shape future research, and a lack of their involvement in formal research and publications could mean that their voices are insufficiently represented. We aimed to modify the well-established Child Health and Nutrition Research Initiative (CHNRI) methodology to address barriers to inclusivity, which are particularly relevant in healthcare that requires highly multidisciplinary care.

**Methods:**

This global research priority-setting exercise for orofacial clefts adapted the CHNRI methodology to include research experts, clinicians from multiple disciplines, and non-technical stakeholders (i.e. patients and parents and non-governmental organisations (NGOs)) on a global basis. A multidisciplinary international steering group proposed and discussed methodological changes to improve inclusivity, including survey edits, subgroups for research questions, a demographics section, translation in French and Spanish, phrasing adaptation, and alternative dissemination techniques.

**Results:**

We received 412 responses and 1420 questions, spanning 78 different countries and 18 different specialties/groups. Challenges remain to improve representation of all groups, with the vast majority of answers (30%) being from surgeons and a comparatively small proportion from patient/parent groups (9%). This also includes managing responses in three languages, effective dissemination, and responses that were not worded as research questions.

**Conclusions:**

This is one of the first CHNRI exercises to involve patients and parents, clinicians, and researchers in its first question submission stage, and the first ever to do so on a global scale. We describe our approach to addressing inclusivity challenges and report related demographic data to serve as a benchmark upon which we hope future CHNRI exercises will improve.

Research prioritisation tools were popularised in recent decades following the 1990 Commission on Health Research for Development (CHRD) landmark report, which highlighted that only 5% of available global health research resources were invested in low-and-middle income countries (LMICs) where 93% of preventable deaths occurred [1]. As a result, tools were developed to reduce global health disparities, especially among resource-poor, but disease-burdened communities. For example, priority-setting tools such as the Essential National Health Research (ENHR) were created to aid researchers to focus on health resources [2]. Their uptake, however, was variable. In 2016, Yoshida et al. [3] conducted a methodical review of priority setting tool uptake in the years following the CHRD report. They found that the ENHR was adopted in only 0.6% of all the sampled exercises from 2001–04. Comparatively, the Delphi and ‘individual consultation’ methods were both popular (24% and 18%, respectively), while the Child Health and Nutrition Research Initiative (CHNRI) emerged as the most accepted one, with a 26% share.

The CHNRI method was developed in 2007 as a transparent framework to conduct research priority-setting exercise [4]. It was built upon ‘crowdsourcing’, whereby the most widely-published researchers within a healthcare field would be invited to submit research questions or ‘options’ they would like to be answered in the future. These research questions/options would be scored against pre-determined CHRNI criteria, including equity, answerability, and the ability to reduce the burden of disease, according to how well each research option would satisfy each criterion. The result is an overall ‘priority score’ for each question on a quantitative scale of 0–100%, which represents the collective optimism of the likelihood that each research question/option would satisfy each priority setting criterion in turn. These scores might then be used by future stakeholders help them determine which areas of research to focus on [5].

To date, there have been no published CHNRI exercises on orofacial clefts (OFC), the most common craniofacial conditions with an incidence of approximately 1 in 700 live births worldwide and significant global variation in the burden of disease, management, and outcomes [6]. Other priority-setting exercises exist, but none on a global scale. These include a UK-wide James Lind alliance focus-group approach [7], a workshop undertaken in 2007 on future research priorities for OFCs [8], a social-media-based research prioritisation for multiple congenital abnormalities [9], a qualitative study on comparing parental and clinical priorities on sleep-disordered breathing in OFCs [10], and a Delphi study on the management of otitis media with effusion in children with cleft palate [11]. In contrast to these approaches, the CHNRI method can be deployed on a global scale, can be completed remotely, considers issues unique to each geographical region, and is adaptable for diverse research areas [12].

Despite this, CHNRI exercises are not without limitations, as they exclusively invite ‘top’ researchers in each healthcare field to submit questions – an approach which has been criticised for bringing bias into the results [13]. They thus exclude clinicians who are not well-published, yet treat high volumes of patients, although they can offer valuable insights into the research priorities that offer the greatest potential impact on improving patient care. This is particularly relevant in LMICs, where this group frequently face many barriers to publishing research.

Past CHNRI processes have already provided lessons regarding their own inclusivity. While the approach recommends including a large and diverse group of stakeholders, including non-professional individuals, their role solely consists of ‘weighing’ pre-defined criteria and setting ‘thresholds’ for a minimum acceptable score against each criterion [14]. In a review of the first 50 CHNRI exercises, Rudan et al. [4] expressed concern regarding the spectrum of research ideas submitted and evaluated in the CHNRI process, observing that it was potentially not comprehensive and that it might overlook some promising research questions. The CHNRI exercises conducted since then have invited experts, some including clinicians, to submit questions [13,15–18], highlighting the importance of seeking opinions from academic, research, programmatic, non-governmental, and donor organisations, both from high-income countries (HICs) and LMICs.

Various recent CHNRI exercises have attempted to include non-technical stakeholders (i.e., patients, carers, families, and representatives) as well. Most notably, a recent CHNRI exercise on musculo-skeletal disorders in the UK gathered question submission from patient groups through a survey advertised in national patient organisations [6]. Another CHNRI exercise involved 25 experts with relevant lived experiences whose questions were gathered through a separate survey [1].

A past OFC prioritisation exercise used the James-Lind Alliance (JLA) approach, which focusses heavily on patient and clinician priorities. This UK-based exercise showed the importance of patient and clinician involvement in priority setting [7]. This pursues the principle of moving away from ‘paternalism’. Promoting patient priorities encourages patient engagement in direct care, and patients receive information about a diagnosis or treatment by a healthcare professional and therefore can voice their preferences, which allows clinicians to combine this with medical and clinical evidence [19]. However, this principle is less popular in policymaking due to factors such as beliefs about patients’ roles, health literary, levels of education, organisational policies, practices, and societal norms [20]. This has taught us that research should be also guided by the priorities and concerns of patients and their families, particularly in conditions that require long-term care and follow up, such as OFC.

Through this CHNRI exercise, we aimed to obtain a representative overview of global research priorities in OFCs by improving inclusivity of under-represented groups. Here we outline the rationale behind the altered CHNRI methodology, how we achieved it, and what some important findings of the process are. While this CHNRI focussed on OFCs, the principles and challenges are relevant to other conditions, particularly those that require highly diverse multidisciplinary team care and where the condition may present additional disease burden in LMICs. Lastly, we aim to report on the results of our approach to improve inclusivity, to serve as a benchmark upon which future CHNRI exercises will improve.

## METHODS

We used a modified CHNRI methodology [4] to improve inclusivity and engagement of multidisciplinary stakeholders globally.

### The steering group

The CHNRI methodology involves selecting a group of process managers who help specify the context in space, i.e., what is known about the problem to be addressed and the values and interests of which stakeholders must be respected when setting research priorities [21]. They also define the scoring criteria that are best linked to the prioritisation exercise that they are performing [5].

We established an international multidisciplinary steering group of 14 technical experts with interests in OFC research. These individuals came from the key disciplines and multiple countries (HICs and LMICs), ensuring a diversity of views from the wider global research community. Their primary role was to comment on the recruitment and data collection tool and to provide general insight on improving inclusivity. This steering group held all meetings virtually, which facilitated multinational collaboration. However, we still recorded all meetings due to differences in time zones and unreliable internet access, while the senior author (FVM) held one-to-one meetings with colleagues outside of the main group meeting. The members, disciplines and countries of the steering group can be found in **Table 1**.

**Table 1:** Steering group members, specialty, and country of residence

Names	Specialty	Country
Felicity V Mehendale	Plastic surgeon	UK
Niki Kouvroukoglou	Medical student	UK
Sanita Sandhu	Foundation doctor (FY1)	UK
Orla Duncan	Psychosocial nurse practitioner	UK
Carrie Heike	Paediatrician	USA
David Fitzimons	Speech and language therapist and data scientist	Australia
Barbara Delage	Nutritionist	France
Gareth Davies	Member of European Cleft Organisation (patient-parent organisation)	France/Netherlands
Debbie Sell	Speech and language therapist	UK
Mekonen Eshete	Plastic surgeon	Ethiopia
Nicola Stock	Research psychologist	UK
Bruce Richard	Plastic surgeon	UK
Marina Campodonico	Paediatric dentist	Chile
Zipporah Gathuya	Anaesthesiologist	Kenya

### Survey design

One researcher (NK) created and refined the survey using the online Joint Information Systems Committee platform, which allows for multiple collaborators, easy editing, easily downloadable data, and is secure. We were also able to design the survey in a colour-blind friendly palette.

Three researchers (NK, SS, and FVM) then presented the CHNRI methodology to the steering group, asking it to consider barriers and propose potential solutions to maximise participation across disciplines and countries. **Box 1** summarises the modifications to increase inclusivity.

Box 1 **Modifications to increase inclusivity in CHNRI Cleft**

1. Separate surveys
  - For technical stakeholders (researchers and clinicians) and non-technical stakeholders (patient-parent organisation and NGO representatives, patients and parents) with consent forms tailored to each group
  - Previous methodology around involvement of stakeholders in the original CHNRI method is described by Kapiriri et al. [14]
2. Changes related to language and phrasing
  - Non-technical language in both surveys to accommodate those without English as their primary language
  - Avoiding the term ‘research question’
3. Translation into French and Spanish.
4. Demographic section data (country of residence and collaborator occupation/group)
  - Rationale: to monitor the representativeness of our respondents. target recruitment and increase responses from diverse groups (**Table 2**)
5. Each submitted response to be matched to a broad subject category and an age group
  - Rationale: to monitor how well each specialty/group was represented (**Table 2**)
6. Dissemination
  - WhatsApp invitations as an additional recruitment method
  - Rationale: WhatsApp is preferred to email communication in some settings countries
7. Social media sharing (Twitter)

The survey and its supplementary documents (participant information sheets, privacy statements, and informed consent forms) were translated into French and Spanish, with separate information sheets and consent forms provided for technical and non-technical collaborators. For this, we employed the help of two native speaker steering group members (BD (French) and MC (Spanish)) and a professional translator (HW – partner coordinator of Translators without Borders). We decided to include French to reach collaborators in France, West-Africa, and Canada, and Spanish to include those from Central/South America. We were also limited in our choice of languages by financial constraints, and we also excluded Asian languages for practical reasons. A Google Translate tool was trialled but abandoned, as it translated specific terms into offensive outdated medical terminology. Similar issues were noticed when the survey was sent to a professional translation service for translation [22].

The landing page of the survey itself offered a choice of language, after which the survey displayed the participant information sheet, a privacy notice, and a consent form to read and sign. There were then two paths to submit questions. This was to satisfy the CHNRI methodology component for scoring research questions in a subsequent survey. Collaborators could choose to participate in this stage for group authorship or they could simply submit questions for no authorship. If choosing the former, the collaborator would click a link to provide their contact details prior to submitting questions. If not, they would progress straight to the question submission page.

On the ‘General participant information’ and ‘Contact details’ sections, collaborators were given the option to identify themselves in a group and/or profession. There were 21 multiple-choice options for occupations/groups in the field of OFC, and collaborators could choose to identify with more than one group (**Table 2**). The steering group unanimously agreed on allowing individuals to select more than one group to accommodate for individuals with multiple expertises in an already highly-multidisciplinary field.

**Table 2:** Multiple choice options within the survey

Occupation/group	Age groups	Patient-centred themes
Anaesthetist	Pre-natal	Breathing problems
Anaesthesia provider	Neonate 0–28 days	Concerns about education/schooling
Audiologist	Infant <28 days and <1 year	Concerns about operation
Community based rehabilitation worker	Toddler 1-3 years	Facial appearance
Dentist	Child >3 years <7 years	Feeding problems
Geneticist	Child 7–12 years	Future children/genetics
Nurse	Teenager 13–19 years	Hearing problems
Nutritionist	20 and more	Mental health issues
Orthodontist	Other	Pregnancy concerns
Oral hygienist		Scar care
Paediatrician		Social issues
Psychologist		Speech problems
Physiotherapist		Teeth problems
Researcher/scientist		Other
Social worker		
Speech therapist		
Surgeon		
Patient with a cleft		
Family member/carer of a patient with a cleft		
Connected with a cleft non-governmental organisation/charity/non-profit		
Other		

On the ‘Question submission’ section, collaborators had to outline their questions or concerns in a large open-ended text box, through which they could submit 1–6 questions. They were invited to submit ‘unanswered questions’, ‘suggestions for quality-of-care improvement’, or ‘concerns and issues with the current level of cleft care’ as opposed to ‘research questions’. We anticipated that some non-clinicians and clinicians who are not engaged in formal research may not feel confident to formulate complete research questions, and that even highly-experienced, highly-engaged clinicians may perceive the term ‘research question’ as being irrelevant to their practice and daily clinical pressures. We, however, aimed to maximise the opportunity to hear from such colleagues, as they are often best able to identify the real-world pressures and challenges to the delivery of safe, high quality, accessible care.

Collaborators were also given the option to select any (or multiple) of 14 patient-centred thematic categories that applied to the question they had written. These were formulated by the steering group and were specific to issues topic areas related with OFCs (**Table 2**). Lay language was used for each category to accommodate non-technical collaborators and was kept broad to reflect the highly multidisciplinary nature of cleft care. The inclusion of a representative from a patient/parent organisation on the steering group from the outset was integral to this approach.

As cleft care extends from the antenatal period to adulthood, collaborators were also given multiple choices when asked to provide applicable age categories. As these needed to be meaningful to a global audience, the steering group defined eight categories adjusted from the WHO guidance [23] (**Table 2**) to truthfully reproduce the lifelong care aspect of OFC in the exercise’s result analysis.

### Collaborator recruitment

**Figure 1:**
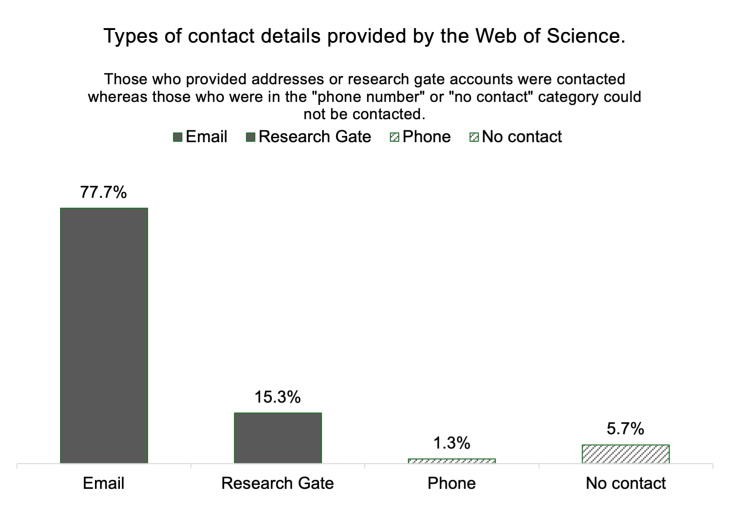
Types of contact details from the 300 most widely published researchers in the OFC field.

The CHNRI method relies on questions submitted from collaborators in a medical field [5,24] and has a well-described approach to selecting the most widely-published 300 researchers in that field based on a Web of Science search. As this allows for a clear method of selection, we followed this step from original CHNRI methodology here and recruited 300 most widely-published OFC researchers. Specifically, the search provided the surnames and initials of these individuals, while we then queried Google, Bing, and PubMed using the formula ‘cleft + surname + initials’ to obtain contact details. In total, 77.7% researchers provided email addresses on published papers, while 15.3% did not. In keeping with our aim to optimise inclusivity, two researchers (NK and SS) searched for alternative ways to contact these authors, using links to ResearchGate accounts where provided. One researcher (NK) contacted each researcher directly through the chat function on ResearchGate; four (1.3%) provided phone numbers as their sole contact method. Lastly, 17 individuals (5.7%) did not display any contact details online (**Figure 1**).

**Figure 2:**
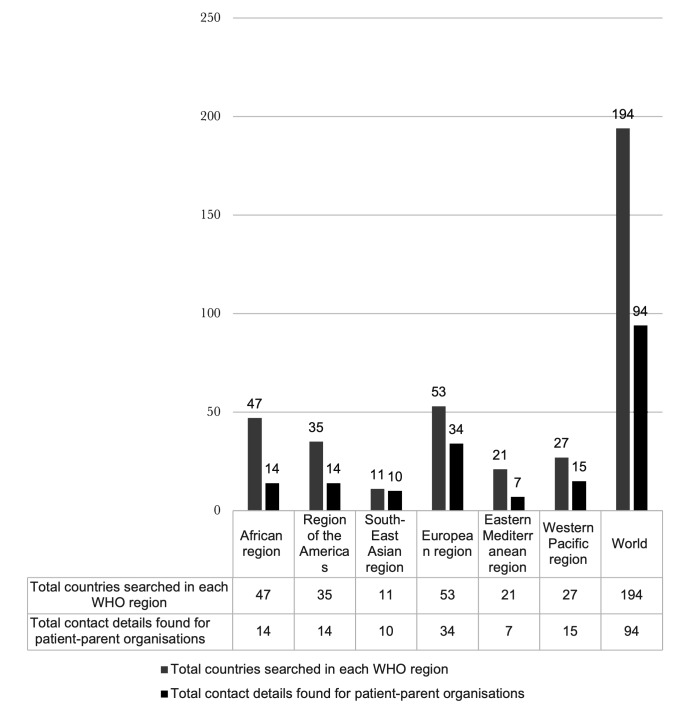
Number of countries searched and the number of patient parent organisations found online for each of the six WHO world regions

However, we also adopted a range of approaches to include clinicians, a broader pool of researchers, NGOs, and national patient/parent organisations involved with any aspect of OFC care or research, as well as any individual or family member affected by an OFC. Accessing contact information for cleft NGOs and national cleft patient-parent organisations was very challenging, as there are no central databases with such details. We included every country in the six WHO regions, and two authors (SS and NK) searched three regions each, querying internet search engines for relevant organisations using the formula ‘Country name +/− cleft NGO +/− patient parent organisation’. Search terms were also translated into French, Spanish, Portuguese, and Arabic to find organisations. A list was compiled which consisted of the country name, the name of a patient parent organisation, and an email/contact number for each. In total, 94 email addresses, 10 phone numbers, and 5 Facebook group links were collected (**Figure 2**).

The survey was disseminated through multiple channels to encourage responses from all target groups. The main method was a mass email containing participant information sheets and informed consent forms sent to addressed obtained from the Web of Science, ResearchGate, and Google searches. We also sent WhatsApp invitations to collaborators in regions where WhatsApp is checked more often than emails for clinical and academic communications, such regions included Africa and South America. The steering group members also shared the survey through their personal and professional social media (Facebook and Twitter) accounts via posters, QR codes, and infographics. As ome members were a part of international cleft organisations, they also promoted the survey through their organisation meetings and mailing lists. Recipients were encouraged to disseminate the survey invitation to colleagues and team members (snowball method). While we recognised that this approach meant we would not be able to determine a denominator to calculate a response rate, our priority was to maximise opportunities for participation.

We anticipated that, despite our efforts to recruit respondents from all disciplines, most collaborators would be surgeons. To increase participation from traditionally underrepresented professions such as nurses, social workers, and other disciplines, one researcher (FVM) requested all surgeons to share the survey with their multidisciplinary team colleagues and to encourage them to participate, and suggested to surgeons that they think more broadly of non-surgical research priorities while submitting in their question.

We monitored the response rates from individual specialties during the survey and tweeted a bar chart showing the disciplines that had engaged with it the most in order to increase participation from underrepresented groups.

### Data management and ethics

The Edinburgh Medical School Research Ethics Committee gave ethical approval for our study (21-EMREC-015), including for the storage and use of personal data on the Joint Information Systems Committee platform and the invitation of collaborators via a WhatsApp link.

On advice from steering group members in Africa and South America, we initially planned to invite collaborators via WhatsApp using a publicly held phone number and give them the option to provide their WhatsApp number on our survey for the future scoring surveys. However, the ethics committee viewed this method as intrusive and/or unprofessional and were reluctant to allow it. We explained that this was the preferred method of contacting researchers in these regions of the world and that we should not allow our own practices in a high-income Western country to discount theirs. It was then agreed that we limit this method of recruitment to researchers only.

## RESULTS

### Overall numbers and global reach

**Figure 3:**
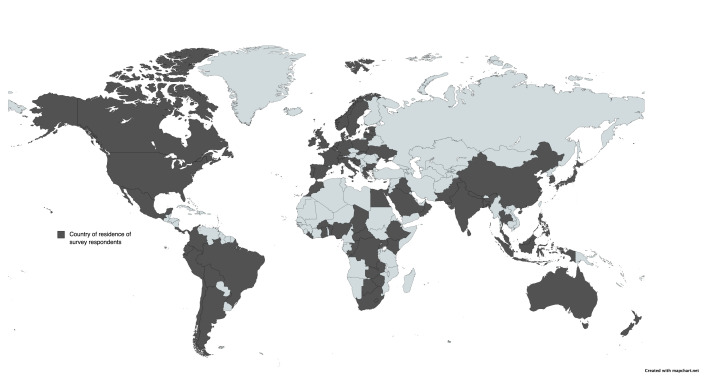
Map of the world showcasing country of residence of collaborators (dark grey). Made via MapChart [30].

Over a period of five and a half months (14 October 2021 to 31 March 2022), we received 412 responses containing 1422 questions. This was over a period of five and a half months (14/10/21 to 31/3/22). We reached 78 countries (at least one collaborator from each country), comprising 40% of countries in the world (**Figure 3**). Approximately 128 (9%) submissions were statements rather than fully formed questions.

### Responses by language and gender

Most collaborators (85.9%) submitted questions in English, and fewer in French (4.1%) and Spanish (10%). The overall gender split as a cohort was 56.57% female vs 43.43% male. The respondents who submitted questions in English and Spanish were predominantly female, while those who submitted in French were mostly males.

### Responses by specialty

The total number of individuals by specialty/group was 480, although we received only 412 responses. This is because collaborators were allowed to choose more than one specialty/group that they belonged to. Surgeons comprised the largest group of collaborators (30.42%), followed by orthodontists (10.21%) and speech therapists (8.54%). Members of patient parent organisations/non-governmental organisations comprised 8.96% of the overall share; they submitted more responses than researchers (7.92%) and dentists (7.71%). There were no responses from French-speaking organisations. Family members of individuals with OFCs comprised 6.67% of the responses (**Table 3**).

**Table 3:** Responses by specialty in each of the three languages

Specialty	English	French	Spanish	Total, n (%)
Surgeon	126	6	14	146 (30.42)
Orthodontist	44	1	4	49 (10.21)
Speech therapist	32	2	7	41 (8.54)
Patient parent organisation/non-governmental organisation	36		7	43 (8.96)
Researcher/scientist	33	1	4	38 (7.92)
Dentist	30		7	37 (7.71)
Family member of individuals with orofacial cleft	27	2	3	32 (6.67)
Anaesthetist	12	2	2	16 (3.33)
Other	12	1	2	15 (3.13)
Nutritionist	10	1	1	12 (2.5)
Paediatrician	9	2	1	12 (2.5)
Geneticist	8			8 (1.67)
Psychologist	8		1	9 (1.88)
Nurse	7	2		9 (1.88)
Patient with a cleft	7		1	8 (1.67)
Social worker	2			2 (0.42)
Audiologist	1			1 (0.21)
General practitioner	1			1 (0.21)
Oral hygienist	1			1 (0.21)
Total				480 (100)

Collaborators belonging to the medical specialties of anaesthesia (3.33%), paediatrics (2.50%), and general practice (0.21%) were underrepresented in the survey. There was only one general practitioner response in English, and none for French and Spanish. We observed a similar pattern for social workers, audiologists, and oral hygienists. Only eight patients with an OFC responded to the survey, comprising 1.67% of the responses. No individuals with OFCs provided responses in French. We also included a ‘other’ group to allow free text responses in case a specialty was missed. This included two otolaryngologists, one plastic surgeon, one obstetrician/anatomy lecturer on congenital anomalies, one paediatric dentist and child psychologist, one dental therapist, one oral surgeon, one paediatric dentist, one academician, one physician’s associate, one carer, and one parent of a child with OFCs.

### Categories of question submission

**Figure 4:**
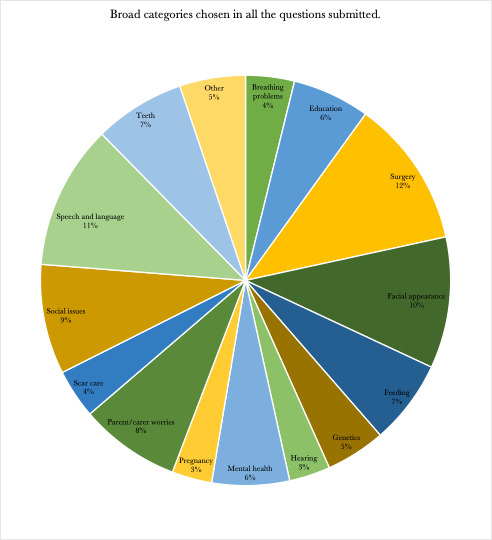
Representation of broad categories in the submitted questions.

Questions about surgery, speech and language, facial appearance and social issues each comprised around 10% of all the questions, with the first being the largest at 12% (**Figure 4**, **Table 4**). The remaining categories each took between 3–8% of the overall share of questions. No groups were significantly more underrepresented than another. The ‘other’ was a free-text question made up 5% of overall question categories. Some of the free-text categorise provided by the participants were ‘aetiology of clefts’, ‘resource management’, ‘all’ (i.e. all of the above), ‘prenatal care counselling’ and ‘quality of life’.

**Table 4:** Broad categories/themes emerging from the question submission

Broad categories	Proportion in all questions, n (%)
Breathing problems	134 (3.84)
Education	213 (6.11)
Surgery	406 (11.64)
Facial appearance	361 (10.35)
Feeding	232 (6.65)
Genetics	164 (4.7)
Hearing	113 (3.24)
Mental health	213 (6.11)
Pregnancy	110 (3.15)
Parent/carer worries	276 (7.92)
Scar care	135 (3.87)
Social issues	302 (8.66)
Speech and language	397 (11.39)
Teeth	250 (7.17)
Other	181 (5.19)
Total	3487 (100)

## DISCUSSION

This is one of the first and largest global CHNRI exercises to invite stakeholders from non-technical backgrounds to propose research ideas. Many previous CHNRI exercises have made efforts to shift their focus to patient and parent involvement within the exercise. Here we aimed to take patient involvement one step further and capture their proposed ideas for future research. We collected 412 responses from individuals in the cleft community, spanning 78 different countries and 19 disciplines and backgrounds, and gathered a total of 1422 questions for future research. This process of enhancing inclusivity of stakeholders from all backgrounds throughout the CHNRI method has had its benefits and its challenges.

### Strengths

We first did not want language to be a barrier in this global exercise. Most global research priority setting exercises have been conducted in English; for example, Irvine et al. conducted their CHNRI exercise in English, French, Spanish and Portuguese [25]. We translated our survey into French and Spanish, as 24 out of 54 African countries are francophone and as Spanish is the most spoken language in Latin America. We also wanted to increase the response number by using native languages and reduce language as a barrier to respondents being able to communicate their thoughts. Sandhu et al. [22] highlighted the importance of native-speaking clinicians checking medical terminology translations, since Google Translate and professional translators may use offensive or outdated terminology [22].

The accessibility the online platform further facilitated a global response and helped collect many responses. Before 2016, most CHNRI exercises emailed Microsoft Excel spreadsheets to participants. We recognised the limitations of this approach for our study, which required wide dissemination by a range of methods, so we opted for an online platform with an easily sharable link. Other CHNRI exercises successfully used web-based platforms such as Inquisim by Cvent [25].

Disseminating the survey to individuals from diverse backgrounds and regions was a crucial step to generating a variety of responses and ensuring inclusivity. We recognised that social media had gained considerable traction in social research, as Facebook, Twitter and YouTube all support participant recruitment and other research activities [9,26]. The benefits are fast dissemination of scientific information to targeted audiences, real-time tracking of recruitment, and cost-efficiency.

A key step in optimising inclusivity is ensuring that invitations to participate are disseminated in formats that are most likely to be noticed by all demographic groups. While email and Twitter are commonly preferred in HIC settings, we recognised that WhatsApp is strongly preferred in some LMICs. Interestingly, our ethics committee expressed concern over our proposed use of WhatsApp, pointing out that this could be ‘intrusive and non-conventional at best, and may be viewed as intrusive or unprofessional by recipients’. We were able to explain and justify the use of WhatsApp and eventually received the ethics committee’s approval. However, this does raise an important point regarding the potential for unconscious bias to lead to exclusion of colleagues from countries that may prefer a form of communication different from the one used in the country in which ethics approval is sought. This underlines the importance of having a diverse international steering committee for global health research projects and suggests the importance of having relevant global research perspectives to inform ethics committee decisions. Other global CHNRI exercises, most of which only used email to communicate with contributors, have mentioned the challenges of acquiring responses from LMIC experts, which highlights the unmet need for varied methods of dissemination of these question submission surveys [18].

### Limitations and lessons on moving forward

Reaching to the patient-parent organisations could only be done via an internet page or online group. Unfortunately, this would mean that organisations without online activity were omitted from this research prioritisation exercise, underlining the importance of having a central global database of all patient parent organisations. We intend to share our search findings to initiate the creation of such a database.

We received 412 responses in total; while this is a lower number than what we were hoping for, it compared well with other global CHNRI exercises that gathered a similar number of responses, such as the one by Tomlinson and colleagues (n = 406) [27]. A reason for fewer responses may have been due to post-pandemic survey fatigue, as survey-based research grew due to pandemic-related data collection limitations [28]. One systematic review in neurosurgery noted that during and after the pandemic, surveys were sent to more geographical regions than before pandemic, but had a lower response rate [29]. In addition, we only translated the survey into only two other languages (Spanish and French), which may have further affected the response rate.

From the total sample, 30.4% of the respondents were surgeons, 8.96% patient-parent organisations, and 1.67% patients. From our steering group’s expert observations, surgeons are often the largest group at international multidisciplinary OFC meetings, with some other specialties being very poorly represented. We anticipated that this would be the case for our study as well and adopted various approaches to improve representation of other disciplines. Despite this, it remains challenging to involve groups that are non-surgical and non-clinical, highlighting how important this effort is, especially in fields with similar multidisciplinary and patient-parent involvement as orofacial clefts.

Notably, 128 (approx. 9.00%) of the submitted questions were not phrased in a traditional research question format, i.e., a coherent sentence followed by a question mark. We believe this is the result of promoting inclusivity and gathering responses from non-research active individuals. It also could stem from encouraging collaborators to submit ‘unanswered questions’, ‘suggestions for quality-of-care improvement’, or ‘concerns and issues with the current level of cleft care’. Examples of non-formed questions include ‘Waiting list’ and ‘My kid has mental delay’. Some submissions also raised topics that appeared to be addressed, to some extent, by existing literature, potentially highlighting the importance of dissemination of research findings.

We will address this during the condensing stage, where the steering group will go through all the responses to merge duplications and similar questions and formulate structured research questions. However, the fact that we were able to reach those who may have little to no formal research involvement and, more importantly, create a setting where they felt able to highlight (albeit briefly) their primary areas of concern demonstrates the importance of improving inclusivity when setting global research priorities.

## CONCLUSIONS

Inclusivity is critically important in global health research when setting research priorities. We have reported our efforts to address this gap, outlined the challenges we faced in doing so, and reported our outcome in terms of the respondents’ demographics and professions.

This was one of the first CHNRI exercises to focus on capturing the opinions of individuals with a condition and those of their relatives and non-research active clinicians from the outset. Perspectives from our international steering group informed all modifications of the CHNRI methodology to optimise inclusivity, and we collected data on respondent demographics to monitor the degree to which we achieved this. We were able to collect responses from geographically and economically diverse settings, as well as large number of disciplines. We also observed that having a native speaker with clinical expertise check all translations is important to ensuring the use of sensitive and appropriate terminology for medical conditions. Using a web-based survey secured access for many who would have faced barriers to join in-person discussions.

Importantly, we found that invitations to participate in global collaborations must be disseminated using a range of methods to avoid a biased sample. It essential to emphasise and justify this to ethics committees to address potential reluctance to approve communication using approaches they may perceive as intrusive. Failure to address this could lead to systematic exclusion of some populations, particularly in LMICs, where WhatsApp is often the preferred method of communication rather than e-mail. We recognise the additional effort required and the difficulties we faced in improving inclusivity as well as the remaining limitations. The patient/parent ‘voice’ was a small percentage of overall responses, and the number of responses from nurses was small compared to the percentage of surgeons. However, measuring and reporting such variations in representation is an important step toward improving inclusivity further so that the underrepresented voices and professions can contribute equitably to research. We hope that publication of the demographics of our collaborators shows the need for ongoing efforts to improve inclusivity, and that our data will serve as a baseline against which future improvements may be evaluated.
